# Sex-Specific Effects of Menaquinone-7 (MK-7) Supplementation on Body Composition and Adiposity Markers

**DOI:** 10.3390/nu18111699

**Published:** 2026-05-27

**Authors:** Rudolf Bittner, Femke de Vries, Francois Machuron, Katarzyna Maresz, Olav Gåserød, Leon Schurgers

**Affiliations:** 1 Department of Biochemistry, Cardiovascular Research Institute Maastricht, University of Maastricht, 6229 ER Maastricht, The Netherlands; rudolf.bittner@maastrichtuniversity.nl (R.B.); femke.devries@maastrichtuniversity.nl (F.d.V.); 2 Lesaffre International, Lesaffre Institute of Science and Technology, 59700 Marcq-en-Baroeul, France; f.machuron@lesaffre.com; 3 Independent Researcher, 30-376 Krakow, Poland; 4 Gnosis by Lesaffre, Research and Development Department, 59700 Marcq-en-Baroeul, France; o.gaserod.ext@gnosis.lesaffre.com

**Keywords:** Vitamin K2, menaquinone-7, BMI, VAT, body composition, menopause, sex specific response

## Abstract

**Background/Objectives:** The benefits of vitamin K are well established in skeletal and cardiovascular health through activation of vitamin K-dependent proteins, including osteocalcin and matrix Gla protein. Emerging evidence also links vitamin K to pathways that influence body composition, including insulin sensitivity, adiponectin regulation, lipid oxidation, and inflammation. Vitamin K deficiency is linked to adverse health outcomes. We therefore evaluated whether menaquinone-7 (MK-7) supplementation alters body composition in adults with low vitamin K status. **Methods**: A total of 243 participants (166 women and 77 men) with low vitamin K status received 180 mcg of menaquinone-7 (MK-7), a form of Vitamin K, daily for one year. Changes in body mass index (BMI), body weight, fat mass, lean mass, and visceral adipose tissue (VAT) were assessed. **Results:** At baseline, higher dp-ucMGP, indicating lower vitamin K status, was positively associated with BMI (r = 0.223; *p* < 0.01), fat mass index (r = 0.200; *p* < 0.01), and VAT (r = 0.286; *p* < 0.001). After one year of supplementation, the total cohort showed a small but significant BMI reduction (−0.66%; *p* = 0.041). Responders (≥183 pmol/L dp-ucMGP drop) showed improvements in fat mass (−1.93%, *p* = 0.039), waist-to-hip ratio (−1.43%, *p* = 0.012), BMI (−1.37% ± 3.21, *p* = 0.004), and weight (−1.25%, *p* = 0.008), while non-responders exhibited an increase in VAT (+7.28%, *p* = 0.002). Women, especially premenopausal ones, experienced greater reductions in BMI, fat mass, and weight. dp-ucMGP levels significantly decreased in 90.9% of women (*p* < 0.001), whereas in men, although 77.8% showed a decrease, the change was not statistically significant. Among high responders (≥183 pmol/L dp-ucMGP reduction), both sexes demonstrated improvement, albeit significant body composition changes were seen only in women. **Conclusions**: Lower vitamin K status was associated with higher BMI, fat mass, and visceral adipose tissue. After one year of MK-7 supplementation, a modest reduction in BMI was observed, with more pronounced improvements in body composition among women. While these sex related differences may suggest that men require higher doses to achieve comparable responses, the current evidence remains preliminary. Further studies with larger cohorts are needed to clarify these findings.

## 1. Introduction

With an ever-increasing rate of obesity within the general population, specifically in the western world, health complications, such as diabetes or cardiovascular disease (CVD) that go along with it, are increasing as well, often related to poor nutrient intake.

Vitamin K, including menaquinones, are linked to increase cardiovascular health and increase insulin sensitivity in Type 2 Diabetes patients [1]. Menaquinones can be specified by MK-n (*n* = 1 to 14), where n stands for the number of repeating isoprenyl units in its side chain. MK-7 has been found to have the longest half-life within the body, rendering it an ideal candidate for supplementation.

This nutraceutical is typically discussed in relation to its cardiovascular protective properties due to a decrease in atherosclerosis and aneurysms because of its activation of matrix Gla protein (MGP), one of several vitamin K dependent proteins (VKDPs). Another VKDP is osteocalcin (OC). Activation of OC plays a role in an increase in adiponectin expression which improves insulin sensitivity levels [2,3].

Vitamin K influences insulin sensitivity through the carboxylation of OC. Carboxylated OC has been linked to improved insulin secretion and sensitivity, although human studies show mixed results [4,5,6,7]. Furthermore, menaquinones (MK-4 [8] and MK-7 [9]) improve mitochondrial function, which is crucial for energy metabolism. It enhances mitochondrial respiratory capacity and biogenesis through the SIRT1 signalling pathway, thereby ameliorating insulin resistance [8,9]. Additionally, MK-7 modulates gut microbiota composition, which in turn affects glycaemic control and insulin sensitivity. This involves increased production of short-chain fatty acids and secondary bile acids, which improve glucose metabolism [1].

Menaquinones have been shown to regulate lipid metabolism, reducing body fat and improving lipid profiles. This is partly due to its role in enhancing adiponectin levels, a hormone involved in regulating glucose levels and fatty acid breakdown [5,10,11]. Also, MK-7 impacts fat metabolism by enhancing fatty acid β-oxidation and regulating genes involved in fat metabolism. This helps in reducing obesity-related metabolic disorders [12,13,14].

MK-7 exhibits antioxidant effects, protecting cells from oxidative stress and apoptosis [9,12,15], and MK has anti-inflammatory properties that contribute to its beneficial effects on metabolic health and chronic diseases [5,16].

Given that MK-7 enhances insulin sensitivity through multiple pathways—including gut microbiome modulation, bone metabolism, lipid regulation, and mitochondrial β-oxidation—while also exerting antioxidant and anti-inflammatory effects, this secondary post hoc analysis aimed to evaluate the impact of MK-7 supplementation on body composition and weight regulation. This work is a complementary analysis of a previous study [17].

## 2. Methods

### 2.1. Study Population

This was a double-blind placebo-controlled clinical intervention trial including 243 volunteers performed by Maastricht University between 2015 and 2018. Prior to participation in the study, written informed consent was obtained from all subjects. As previously outlined, the following details have been provided: information pertaining to the recruitment process, the method of randomisation, and the participants [18]. The subjects of this study were aged between 40 and 70 years, had a BMI between 20 and 35 kg/m^2^, were of Caucasian race, and had circulating dp-ucMGP > 400 pmol/L. Excluded were subjects suffering from cardiovascular disease, hyperlipidaemia, a blood coagulation disorder, a history of gastrointestinal or metabolic disease, the use more than 3 units of alcoholic beverages per day, and the use of oestrogen replacement, corticosteroids, anticoagulants, or vitamin K-containing dietary supplements. The conducted study (NCT02404519) is in accordance with the guidelines in the Declaration of Helsinki and all procedures regarding human subjects were approved by the Medical Ethics Committee of the Maastricht University (Maastricht, The Netherlands).

### 2.2. Compliance

Compliance with the protocol was monitored by counting the remaining tablets after 6 months and at the end of the study. The adherence to the protocol for the MK-7 group was 96.3%, and for the placebo group it was 97.5%.

### 2.3. Study Design

After randomization, participants received capsules with 180 mcg MK-7 (MenaQ7, Nattopharma AS, Oslo, Norway—now part of Gnosis by Lesaffre, Marcq-en-Barœul, France) (*n* = 121) or a matching placebo (*n* = 122) to be consumed once a day during breakfast or dinner. During the 12-month study period, the subjects were requested to adhere to their habitual dietary regime. Blood was taken at intake (inclusion/exclusion), at baseline and at the end of the study by venepuncture to prepare EDTA-plasma. Of the 166 women that participated in the clinical study, a total of 165 women where selected and divided into pre/peri-menopausal women (*n* = 78) and post-menopausal women (*n* = 87) subgroups. The exclusion of one participant was necessitated by the absence of confirmation of post-menopausal status. The onset of menopause in post-menopausal women was determined to be 12 months subsequent to the final menstrual period.

### 2.4. Biomarkers

Dp-ucMGP plasma levels were measured using an ELISA test as described before [19]. Intra- and inter-assay variations were 7.6 and 6.8%, respectively, and the lower detection limit was 50 pmol/L. Paired samples (at baseline and endpoint plasma samples) of each subject underwent assessment on the same ELISA plate in order to minimise inter-assay variation. Responders of the MK-7 supplementation are defined by a decrease of −183 dp-ucMGP pmol/L.

### 2.5. Blood Sampling

Non-fasting blood was taken at the intake visit for the preparation of EDTA plasma (4 mL blood) in order to determine circulating dp-ucMGP levels. For the study visits at baseline and after 1 year of treatment fasting, blood samples were taken for the preparation of citrated plasma (10 mL blood), EDTA-plasma (10 mL blood), serum (10 mL blood), and NaF- plasma (5 mL blood). In total, 35 mL blood will be taken. The samples were collected in sterile dry glass tubes (10 mL in Greiner, Vacuette, Kremsmünster Austria; 5 mL in BD Vacutainer, Becton Dickinson, Franklin Lakes, NJ, USA). For serum preparation, the blood was allowed to clot for 30 min at room temperature and subsequently centrifuged for 10 min at 2500× *g*. Plasma was prepared immediately after blood taking by centrifugation for 10 min at 2500× *g*. The serum and plasma were subsampled in small aliquots and immediately frozen at −80 °C until use.

### 2.6. Anthropometrics

At the intake visit, at baseline and after 1 year, body height was measured to the nearest 0.5 cm using a wall-mounted stadiometer. Body weight was measured to the nearest 0.1 kg, with participants wearing light clothing and no shoes. BMI was calculated as body weight (kg) divided by the square of height (m^2^). Waist and hip circumference were measured with a precision of 0.5 cm. Waist-to-hip ratio was calculated as the waist circumference divided by the hip circumference.

### 2.7. Total Body Scan with DXA

To determine indices for fat mass (Fat Mass Index, FMI), lean mass (Fat Free Mass Index, FFMI), and visceral adipose tissue (VAT), a total body scan has been performed by dual energy X-ray absorptiometry (DXA) using a Hologic Discovery-A (Holigic Inc., Waltham, MA, USA). The total body scans were performed at baseline and after 1 year of treatment, with reported coefficients of variation typically below 2% for fat mass and lean mass.

### 2.8. Statistical Analyses

The baseline parameters of the two groups of menopausal women (pre-/peri- versus post-menopausal) were compared using T-tests in the event of data normality. When this assumption was rejected, Wilcoxon signed tests were used.

The progression of cardiovascular parameters between the initial baseline and the final timepoint (12 months of supplementation with placebo or MK-7) was calculated using the relative difference from the initial baseline (expressed in percentage).

The impact of MK-7 supplementation in comparison to a placebo was analysed using Analysis of Variance (ANOVA) models with menopausal status and treatment as the primary factors and the interaction term to ascertain if the treatment effects were heterogeneous within the two menopausal groups. The assumptions underlying the analysis were examined to ensure the validity of the statistical methods employed. Specifically, the normality of the residuals and the equality of the variances were investigated. In the event of a violation of one of these assumptions, the data were log-transformed using the following formula, with min denoting the minimum to consider negative values. Treatment effects on the evolution of cardiovascular parameters within the menopausal groups were evaluated using adjusted *p*-values (employing the Benjamini–Hochberg method to control the false discovery rate). The same analysis was performed for the study of the stiffness index (SI) group effects on the cardiovascular parameters within the two treatment groups. In pursuit of this objective, the entire cohort of patients was stratified into two distinct groups, contingent on the position of their stiffness index at the baseline measurement point, as compared with the overall median SI (9.83).

The analyses were performed using R statistical programming language version 4.4.2 (R Foundation for Statistical Computing, Vienna, Austria). The various characteristics and their respective abbreviations are described in the Abbreviations Section, with the relevant equations provided for those cases where they are applicable.

## 3. Results

This double-blind placebo-controlled clinical intervention trial included 243 people, with a dp-ucMGP level >400 pmol/L at baseline in the age range 40–70. Excluded was one woman with missing menopausal status information.

### 3.1. Poor Vitamin K Status Is Positively Correlated with BMI, VAT and Fat Mass Index at Baseline

**Figure 1:**
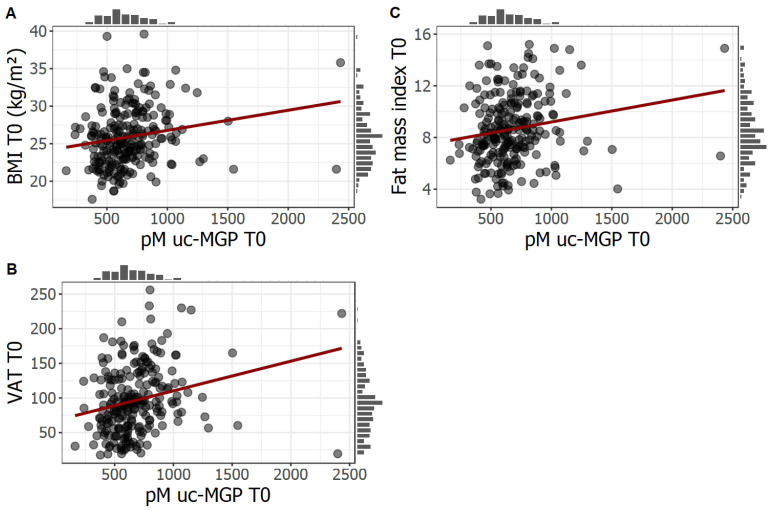
Low vitamin K status is positively correlated with BMI, VAT, and fat mass index. Spearman correlation for (**A**) BMI (0.23; *p* < 0.001), (**B**)VAT (0.286; *p* < 0.001), and (**C**) fat mass index (0.2; *p* = 0.002).

Baseline low vitamin K status was positively correlated with BMI (0,23; *p* < 0.001), VAT (0.286; *p* < 0.001) and Fat mass Index (0.2; *p* = 0.002) (Figure 1).

### 3.2. MK-7 Supplementation Decreases BMI

After one year, a significant decrease in BMI of −0.66% (*p* = 0.041) was observed in the group supplemented with MK-7, unlike the placebo group (not significant decrease). (Table 1).

### 3.3. Women, Especially Pre+Perimenopausal Women Benefit from MK-7 Supplementation

When comparing pre+perimenopausal women (*n* = 34) with postmenopausal women (N = 48), no significant results were observed. However, within the pre+perimenopausal group, a significant decrease over time in weight (−1.36%, *p* = 0.039) and in BMI (−1.61%, *p* = 0.02) was observed (Table 2).

### 3.4. MK-7 Supplementation Responders Show Significant Increases in Their Body Composition Markers

Responders of the MK-7 supplementation showed significant improvements in body composition. Their fat mass decreased by −1.93% ± 6.44 (*p* = 0.039). Furthermore, their BMI was reduced by 1.37% ± 3.21 (*p* = 0.004), as did their waist to hip ratio by −1.43% ± 3.86 (*p* = 0.012) and their weight by −1.25% ± 3.20 (*p* = 0.008). In the non-responder group, we saw an increase in VAT over time by 7.27% ± 18.1 (*p* = 0.002). Comparing these groups, we observed significant differences in VAT (+7.27% ± 18.1 vs. −2.34% ± 16.7; *p* = 0.004), Fat mass (+1.81% ± 9.45 vs. −1.93% ± 6.44; *p* = 0.014), Waist to hip ratio (+0.64% ± 5.22 vs. −1.43% ±3.86; *p* = 0.017), and weight (+0.05% ± 3.47 vs. −1.25% ± 3.20; *p* = 0.042) (Table 3).

### 3.5. Predominantly Women Are Responders and Benefit in Their Body Composition with MK-7 Supplementation

Women represented 76% of all responders (*n* = 37 out of 50) and showed decreased fat mass over time (−2.89% ± 6.72) which was significantly different (*p* = 0.041) from the one in Men subgroup (+0.79% ± 4.77) (Table 4).

## 4. Discussion

In this study, we show that MK-7 supplementation can significantly alter body composition. Overall, a reduction in BMI in the MK-7 supplementation group was observed. Further subgroup analysis revealed a gender specific effect, revealing women benefited significantly in their body composition through MK-7 supplementation. Subgroup analyses revealed that pre- and perimenopausal women had a favourable change in their body composition. Additionally, MK-7 supplementation responders also showed significant improvements in body composition, especially female high responders benefitted.

No lifestyle and calorie intake interventions were taken, yet significant changes in body composition were observed. It has been observed that minor alterations in weight, fat mass or BMI of around 1–2% may indicate the potential effectiveness of MK-7 supplementation, even without deliberate effort. This suggests that combining MK-7 with targeted weight loss programmes in vitamin K deficient persons could help people achieve a healthier body composition.

Research on menaquinones and body composition demonstrates mixed outcomes, albeit with limited overall significance. Tarkesh et al., 2020 found a positive effect on body composition, glycaemic indices, and lipid profile in polycystic ovary syndrome patients receiving 90 µg MK-7 daily [20]. However, Karamzad et al. in 2022 saw no effect on body composition in type 2 diabetes patients [21]. In this study, older adults with a BMI 27–35 were observed for 12 weeks, a challenging timeframe to observe body composition changes. In the aforementioned study, MK-7 supplementation improved PIVKA-II, although the key extra-hepatic marker dp-ucMGP did not differ vs placebo after adjustment indicating that vitamin K-dependent changes most relevant to extra-hepatic tissues may have been insufficient [21]. The study from Yu Sun and colleagues showed modest reductions in weight, body fat, and waist circumference. Participants received 100 µg of MK-7; however the study does not specify which form of MK-7 was supplemented [22]. MK-7 was one component of the formulation of the formulation, which comprised vitamin D3, MK-7, B6, B12, and magnesium. This fact renders the interpretation of the results somewhat challenging.

It is important to note that specific populations may exhibit differential responses. In postmenopausal women, a 3-year MK-7 intervention revealed no overall effect on body composition. However, “good responders” with strong osteocalcin carboxylation increases showed reduced abdominal and visceral fat [11]. Cross-sectional analysis demonstrated that higher vitamin K status correlated with lower body weight, BMI, and trunk fat mass, with long-term supplementation helping to maintain stable weight in comparison to placebo groups that gained weight [23]. In this study, the responder effect was similar, finding positive changes in body composition. In contrast, patients undergoing haemodialysis exhibited an inverse correlation between MK-7 levels and BMI, with overweight and obese patients exhibiting significantly lower MK-7 concentrations [24]. Two studies investigated dp-ucMGP and adiposity and observed a correlation between MGP and obesity/adiposity, respectively [25,26]. Additionally, Shea and colleagues linked greater adiposity to poorer circulating vitamin K status [27].

In summary, the evidence on MK-7 and body composition is mixed and likely population-specific. It is important to note that trials used different measurement methods, DXA and bioelectrical impedance analysis. MK-7 related changes in fat distribution appear method and population, dose and duration dependent, and baseline vitamin K status may also play a role.

Across different populations, as also shown in this publication, low vitamin K status correlates with a higher adiposity status. There are several possible explanations for this phenomenon. A lower vitamin K status contributes to an unhealthy fat distribution, adiposity lowers circulating vitamin K-dependent biomarkers, or, since higher adiposity is associated with worse diet habits, these individuals do not receive adequate nutritional intake of vitamin K [28]. Supplementing with MK-7 is a valid strategy to ensure adequate vitamin K status.

Adeli and colleagues showed that MK-7 supplementation positively affected fasting blood sugar and leptin levels but not adiponectin. However, no responder effect has been observed here [29]. Furthermore, Qu and colleagues observed MK supplementation in C. elegans enhances fat degradation and digestion [12]. However, animal studies are not necessarily translatable to the human body.

From a mechanistic point of view, Ferroptosis Suppressor Protein 1 (FSP1) could play a role in fat metabolism. FSP1, a recently discovered protein in the vitamin K cycle, is highly expressed in fat tissue [30]. In their review from 2022, Zhang et al. discuss how ferroptosis increases obesity [31].

Nuclear Factor Erythroid 2-Like 2 (NRF2) is the transcription factor for FSP1. Menaquinone supplementation has been shown to increase NRF2 levels, due to a decrease in reactive oxygen species (ROS). It could be, due to Vitamin K supplementation, that the high expression levels of FSP1 in adipocytes is altered. This could lead to a change in ferroptosis and decrease in adipose tissue, as we have observed in this study. Further research is warranted to assess the impact of MK-7 supplementation on ferroptosis and adipose tissue development in humans. MK-7 supplementation has been associated with favourable shifts in markers of body composition, and previous studies have suggested potential direct benefits of menaquinones for cardiovascular health [17,32]. While these observations raise the possibility that improvements in body composition could contribute indirectly to cardiovascular outcomes, the current evidence remains preliminary. More rigorous and adequately powered studies are needed to clarify the extent to which MK-7 influences body composition and to determine whether such effects translate into meaningful cardiovascular benefits.

## 5. Conclusions

In conclusion, a poor vitamin K status was associated with higher BMI, fat mass, and visceral adipose tissue. Optimising vitamin K status, achieved through dietary modification or supplementation with MK-7, led to consistent improvements in vitamin K-dependent biomarkers. The modest effects in reducing BMI, observed predominantly in women, and fat distribution are population-dependent and require further studies with larger cohorts to clarify these findings.

## Figures and Tables

**Table 1:** MK-7 supplementation impact on body measurements.

Relative Change from Baseline (%) Parameter	Placebo N = 122			MK-7 N = 121		
	Mean ± SD ^1^	95% CI ^2^ of the Mean	*p* ^3^	Mean ± SD ^1^	95% CI ^2^ of the Mean	*p* ^3^
Visceral adipose tissue	−1.58 ± 19.94	−5.21; +2.06	0.39	+3.18 ± 17.88	−0.09; +6.46	0.057
Fat Mass	+0.80 ± 10.0	−1.02; +2.62	0.39	+0.27 ± 8.31	−1.25; +1.79	0.73
Lean Mass	−0.28 ± 5.69	−1.31; +0.76	0.60	−0.31 ± 2.37	−0.75; +0.12	0.16
Body Mass Index	−0.43 ± 4.48	−1.25; +0.38	0.30	**−0.66 ± 3.45**	**−1.29; −0.03**	**0.041**
Waist to hip ratio	−0.76 ± 5.30	−1.73; +0.21	0.12	−0.29 ± 4.72	−1.15; +0.58	0.51
Weight	−0.25 ± 4.28	−1.04; +0.53	0.52	−0.46 ± 3.38	−1.08; +0.16	0.14
Bone mineral density	−0.33 ± 5.94	−1.42; +0.75	0.54	−0.39 ± 5.18	−1.34; +0.56	0.42

^1^ SD: Standard-Deviation; ^2^ CI: Confidence Interval; ^3^ *p*: *p*-value of significance of nullity of the mean. Bold values indicate *p* < 0.05.

**Table 2:** Menopausal status impact. Body measurements of pre+perimenopausal (N + 34) compared with postmenopausal (N = 48) people within the MK-7 supplementation group.

Relative Change from Baseline (%) Parameter	Pre + Peri N = 34			Post N = 48		
	Mean ± SD ^1^	95% CI ^2^ of the Mean	*p* ^3^	Mean ± SD ^1^	95% CI ^2^ of the Mean	*p* ^3^
Visceral adipose tissue	−0.36 ± 19.6	−7.19; +6.45	0.91	+3.73 ± 16.6	−1.13; +8.59	0.13
Fat Mass	−2.10 ± 7.02	−4.55; +0.35	0.090	−0.12 ± 6.61	−2.06; +1.82	0.90
Lean Mass	−0.54 ± 2.50	−1.41; +0.34	0.22	−0.56 ± 2.17	−1.20; +0.08	0.085
Body Mass Index	**−1.61 ± 3.83**	**−2.94; −0.27**	**0.020**	−0.66 ± 3.15	−1.59; +0.26	0.16
Waist to hip ratio	−1.11 ± 4.11	−2.54; +0.33	0.13	−0.46 ± 5.21	−1.99; +1.07	0.55
Weight	**−1.36 ± 3.68**	**−2.65; −0.07**	**0.039**	−0.66 ± 3.11	−1.58; +0.25	0.15
Bone mineral density	−0.04 ± 1.71	−0.63; +0.56	0.90	−1.03 ± 7.92	−3.35; +1.30	0.38

^1^ SD: Standard-Deviation; ^2^ CI: Confidence Interval; ^3^ *p*: *p*-value of significance of nullity of the mean. Bold values indicate *p* < 0.05.

**Table 3:** Responder impact within the MK-7 subpopulation *.

Relative Change from Baseline (%) Parameter	Non-Responders N = 63			Responders N = 50		
	Mean ± SD ^1^	95% CI ^2^ of the Mean	*p* ^3^	Mean ± SD ^1^	95% CI ^2^ of the Mean	*p* ^3^
Visceral adipose tissue	**+7.27 ± 18.1**	**+2.74; +11.9**	**0.002**	−2.34 ± 16.7	−7.08; +2.40	0.33
Fat Mass	+1.81 ± 9.45	−0.57; +4.19	0.13	**−1.93 ± 6.44**	**−3.76; −0.10**	**0.039**
Lean Mass	−0.25 ± 2.48	−0.87; +0.38	0.43	−0.48 ± 2.29	−1.13; +0.18	0.15
Body Mass Index	−0.22 ± 3.60	−1.13; +0.69	0.63	**−1.37 ± 3.21**	**−2.29; −0.46**	**0.004**
Waist to hip ratio	+0.64 ± 5.22	−0.67; +1.96	0.33	**−1.43 ± 3.86**	**−2.53; −0.33**	**0.012**
Weight	+0.05 ± 3.47	−0.83; +0.92	0.91	**−1.25 ± 3.20**	**−2.15; −0.34**	**0.008**
Bone mineral density	−0.87 ± 6.83	−2.59; +0.85	0.32	+0.29 ± 1.92	−0.26; +0.83	0.30

^1^ SD: Standard-Deviation; ^2^ CI: Confidence Interval; ^3^ *p*: *p*-value of significance of nullity of the mean; * responders defined by a change of −183 dp-ucMGP pmol/L. Bold values indicate *p* < 0.05.

**Table 4:** Gender impact within the responder group (N = 50).

Relative Change from Baseline (%) Parameter	Men N = 13			Women N = 37		
	Mean ± SD ^1^	95% CI ^2^ of the Mean	*p* ^3^	Mean ± SD ^1^	95% CI ^2^ of the Mean	*p* ^3^
Visceral adipose tissue	−0.71 ± 9.26	−6.31; +4.89	0.79	−2.91 ± 18.7	−9.14; +3.32	0.35
Fat Mass	+0.79 ± 4.77	−2.09; +3.67	0.56	**−2.89 ± 6.72**	**−5.13; −0.65**	**0.013**
Lean Mass	+0.20 ± 2.67	−1.42; +1.81	0.79	−0.71 ± 2.14	−1.42; +0.00	0.050
Body Mass Index	−0.44 ± 2.52	−1.96; +1.09	0.54	**−1.70 ± 3.39**	**−2.83; −0.57**	**0.004**
Waist to hip ratio	−0.89 ± 4.36	−3.53; +1.74	0.47	**−1.62 ± 3.72**	**−2.86; −0.38**	**0.012**
Weight	+0.06 ± 2.65	−1.54; +1.66	0.94	**−1.70 ± 3.28**	**−2.80; −0.61**	**0.003**
Bone mineral density	+0.39 ± 1.60	−0.57; +1.36	0.39	+0.25 ± 2.04	−0.43; +0.93	0.46

^1^ SD: Standard-Deviation; ^2^ CI: Confidence Interval; ^3^ *p*: *p*-value of significance of nullity of the mean. Bold values indicate *p* < 0.05.

## Data Availability

The original contributions presented in this study are included in the article. Further inquiries can be directed to the corresponding author.

## Abbreviations

The following abbreviations are used in this manuscript:

MK-7	Menaquinone-7
MK	Menaquinone
VAT	Visceral adipose tissue
BMI	Body Mass Index
CVD	Cardiovascular Disease
MGP	Matrix Gla Protein
VKDP	Vitamin K dependent Protein
OC	Osteocalcin
dp-ucMGP	dephosphorylated-uncarboxylated Matrix Gla Protein
FMI	Fat Mass Index
FFMI	Fat free mass index
DXA	X-ray absorptiometry
ANOVA	Analysis of Variance
SI	Stiffness Index
FSP1	Ferroptosis Supressing Protein 1
NRF2	Nuclear factor Erythroid 2 -like2
ROS	Reactive Oxygen Species
